# Property Taxes and Growth Patterns in China: Multiple Causal Inference Methods

**DOI:** 10.3389/fpsyg.2022.919428

**Published:** 2022-07-05

**Authors:** Hejie Zhang, Shenghau Lin

**Affiliations:** ^1^School of Economics and Management, Zhejiang Sci-Tech University, Hangzhou, China; ^2^Department of Public Administration, Law School, Ningbo University, Ningbo, China

**Keywords:** property taxes, house prices, growth patterns, DID, PSM-DID, a panel data approach for program evaluation

## Abstract

According to neoclassical growth theory, there are two main patterns of economic growth, namely, intensive growth, which depends on total factor productivity (TFP), and extensive growth, which relies on factor input. This study explores the impacts of property taxes on growth patterns by considering the property tax pilots in Shanghai and Chongqing as a quasi-natural experiment. For evaluation, we applied multiple causal inference methods, including DID, PSM-DID, and a panel data approach for program evaluation. We found that the pilot of Shanghai contributed to intensive growth, while the pilot of Chongqing reinforced the prevailing extensive growth. Specifically, Shanghai's property taxes restricted the buying of multiple homes and oversized homes, thereby reducing house prices and increasing TFP. Chongqing's property taxes are mainly for high-end houses, causing the substitution effect between high-end homes and ordinary houses; thus, the pilot increased the prices of ordinary houses and the average house price, which stimulated factor input and economic growth but decreased TFP. This study provides empirical evidence of the causal relationships between property taxes and growth patterns, indicating that transitional economies should avoid narrow tax bases during property tax reform for intensive growth.

## Introduction

According to neoclassical growth theory (Solow, 1956; Barro and Sala-i-Martin, 1991), economic growth is determined by labor input, capital input, and total factor productivity (TFP). TFP refers to the residual value excluding the contribution of labor and capital to economic growth, which stems from technological progress and efficiency improvement. Because of a diminishing marginal return on capital, TFP is the only factor determining long-term economic growth and is widely considered the leading indicator of the quality and sustainability of economic development.

It has been shown that labor and capital are the most crucial factors driving China's economic growth (Zhang et al., 2021). Surplus labor supply has led to low wages, and continuous investments have led to low returns on capital, severe environmental pollution, and huge financial risks. Therefore, this extensive growth pattern relying on factor input must change to an intensive growth pattern for continuous economic development. The transformation of China's economic growth is inseparable from the intensive growth of the real estate market, as this industry has been an engine of growth in China. According to China's National Bureau of Statistics, real estate investments increased by 21.3% per year from 2000 to 2019, which accounted for about 20% of social fixed asset investments. However, the rapid growth of the real estate industry relies on the massive input of capital, labor, and other factors rather than TFP (Campello et al., 2010; Rabe and Taylor, 2012; Chen et al., 2015). This does not support intensive growth.

Property taxes represent a fundamental policy tool for the promotion of intensive growth in the real estate market. On 23 October 2021, the Standing Committee of the National People's Congress of China authorized the State Council to pilot property taxes in selected cities. As China has not levied property taxes on a national scale, it is not possible to measure their impact on intensive growth. However, as early as 2011, the Chinese government implemented property taxes in Chongqing and Shanghai. These cities are municipalities that fall directly under the central government, and their house prices are representative of prices country-wide, house prices in Shanghai are among the highest in the country, and those in Chongqing are average. Therefore, these pilot cities provide excellent research objects. An evaluation of the impact of property taxes in China is essential to upcoming property-tax reform. Evidence of the heterogeneous impacts of different property tax policies on house prices, economic growth, and TFP will serve as valuable references for the design of future policy in China. Our results also have reference value for other developing countries aiming to facilitate intensive economic growth.

Economic growth is determined by factor input and TFP. We found that Shanghai's property taxes slowed economic growth but significantly increased TFP by reducing house prices. Therefore, the positive effect of improving TFP could not offset the negative impact of decreasing factor input. Thus, even as TFP improved, economic growth slowed down. Chongqing's property taxes were mainly for high-end houses rather than for ordinary houses. The pilot tax policy encouraged buyers who might have been considering high-end homes instead of purchase ordinary houses, which increased the prices of ordinary houses and the average house price. Thus, Chongqing's property taxes pilot stimulated economic growth but decreased TFP. Therefore, the positive effect of increasing factor input offset the negative impact of decreasing TFP. Thus, even as TFP fell, Chongqing's economic growth accelerated. In sum, the pilot in Chongqing strengthened extensive growth patterns while the pilot in Shanghai contributed to intensive growth. With high-quality development as their goal, the Chinese government should perfect property tax policies to increase TFP when the economy is stable or booming, in order to achieve sustainable development without causing a severe economic downturn.

A few studies exist on the impact of property taxes on growth patterns. This study explores the impact by considering the real estate policy experiments in Shanghai and Chongqing and multiple causal inference methods. Evidence of the causal relationships between property taxes and growth patterns represents the main contribution of this study. Our findings have reference value for transitional economies beyond China for the effective regulation of the real estate market and the promotion of intensive growth through property tax reform.

The remainder of this study is structured as follows. The next section introduces the property tax pilots in Shanghai and Chongqing. Based on the relevant literature, we hypothesized their effects on growth patterns. Subsequently, our methodology, including the empirical model, method, and data collection are discussed. Our results are presented, followed by our conclusions and policy implications.

## Literature, Institutional Background, and Hypothesis Development

Existing studies on the impacts of property taxes on house prices have yet to reach a consensus. Some studies claim that property taxes increase house prices (Coombs et al., 2012; Liberati and Loberto, 2019). Although real estate developers will not bear any tax as capital can flow freely, buyers will. Moreover, buyers will make a trade-off between house prices and public services, and property taxes may increase the house prices by funding public spending (Arik, 2021; Carrillo et al., 2021). Other researchers have reported that property taxes can effectively decrease house prices (Angjellari-Dajci et al., 2015; Oliviero et al., 2019; Bø, 2020; Li et al., 2020; Zhu and Johnson, 2020; Giertz et al., 2021). The capitalization equation clearly illustrates the mechanism underlying this effect:


(1) $$\begin{array}{l} p_{t}=\overset{n}{\underset{s=t}{\sum }}\frac{\left(y_{s}-\tau p_{S}\right)}{{\left(1+i\right)}^{s-t}}, \end{array}$$

where *p*_*t*_ is the house prices in period *t* (1 ≤ *t* ≤ n), *y*_*s*_ is the real estate value in period s, *i* is the interest rate, and τ is the tax rate. Obviously, *p*_*t*_ decreases as τ increases.

Another perspective is that different property tax policies have heterogeneous impacts on house prices (Bai et al., 2014; Du and Zhang, 2015). Although Chongqing and Shanghai simultaneously started property tax pilots in 2011, their policies have differed significantly. First, Shanghai only taxed newly-purchased houses, while Chongqing taxed both newly bought and existing homes. Property taxes in Chongqing were focused on large-area and high-priced houses, with no tax for small-area and low-priced houses. Second, Chongqing treated immigrants and residents equally, while Shanghai was favorable to residents, reflecting Shanghai's intention to limit the inflow of migrants. Shanghai's tax objects included houses newly purchased by migrant families, but only freshly-purchased second and above houses were taxed for residents. Third, Chongqing's property taxes were higher than those in Shanghai. Rates in Shanghai were 0.4–0.6%, while those in Chongqing were 0.5–1.2%. Moreover, Shanghai levied taxes with a 70% discount on the tax payable, while Chongqing had no discount. Finally, there were differences in tax exemption. Shanghai multiplied the family population by 60 m^2^ as a tax-free area. In Chongqing, the tax-free area of already-purchased single-family houses was 180 m^2^, while that of newly-purchased single-family houses and high-end houses was 100 m^2^ (refer to Table 1 for details).

**Table 1:** Rules for property-tax pilots.

	**Shanghai**	**Chongqing**
Regions	Whole city	Nine main urban areas
Objects	Houses newly-purchased by migrant families; newly purchased second and above houses by local families	Large-area and high-priced houses
Tax rates	0.4 or 0.6%	0.5–1.2%
Discounts	70% of the tax payable	No discount
Tax-free areas	Less than 60 m^2^ per capita	180 m^2^ for existing single-family houses; 100 m^2^ for newly-purchased single-family homes and high-end houses

Bai et al. (2014) found that property taxes lowered Shanghai's average house price by 11–15% but increased Chongqing's average home price by 10–12%. The effect of Shanghai's property taxes is explained by Equation (1). In addition, Shanghai, as China's economic and financial center, has a special economic status. There has been enormous demand for real estate investment and speculation before the property tax pilot. After the pilot, however, holding costs increased for investors with many houses in Shanghai, and their investment demand decreased, resulting in a decline in house prices.

Why did house prices increase in Chongqing? The tax focus on large-area and high-priced houses may have introduced the substitution effect, which caused those looking at high-end houses to purchase ordinary homes. Residents may have also been concerned that ordinary houses would be taxed in the future, which may have pushed up ordinary house prices. According to the Chongqing Municipal Bureau of Land, Resources, and Housing, 3 months after the property taxes were introduced, transactions involving houses of <100 m^2^ increased by 20%. Notably, 10 months after the launch of the pilot, the growth rate reached 17.8%, and transactions involving ordinary houses in the main urban areas accounted for total sales of 93.2%. As ordinary houses occupied the majority of the housing market, these changes increased the average house price. Therefore, this study puts forward our first hypothesis:

H1*: Chongqing's property tax pilot increased ordinary house prices and the average price, while Shanghai's property tax pilot reduced house prices*.

Increased house prices promote economic growth by stimulating investment and labor input (Kishor, 2007; Miller et al., 2011; Cai et al., 2020). If Chongqing's property tax pilot increased house prices, it might promote economic growth but might reduce TFP. This is because, high house prices attract capital flow into the real estate industry (a high-profit low-production industry), drawing funds away from high-tech, high-production industries.

Furthermore, workers are likely to demand a pay increase to cover increasing housing costs, which will further increase the expenses of high-production enterprises (Rabe and Taylor, 2012; Liu and Yang, 2020). The bubble created by the real estate market may also cause enterprises to allocate more resources to the real estate industry (Campello et al., 2010; Chen et al., 2015), which may have nothing to do with their core business. Indeed, 40% of listed companies in China have real estate investments (Chen and Wen, 2017). As a consequence, resource allocation becomes inefficient. Moreover, the rapid development of the real estate industry and high house prices will cause huge financial risks (Bullard et al., 2009; Tajik et al., 2015; Gonzalez et al., 2018, 2021; Wegener et al., 2019).

Finally, the real estate industry consumes enormous amounts of energy and causes severe pollution alongside low energy efficiency, particularly by reinforcement and cement firms (Chen et al., 2019). The above reasons cause high house prices to exert a short-term positive effect on economic growth but a negative effect on the TFP and sustainable development of the economy (Gao and Dong, 2020; Yang and Pan, 2020).

Our first hypothesis suggests that Shanghai's property tax pilot should improve TFP, but what about its effect on factor input and economic growth? Therefore, this study puts forward the next two hypotheses:

H2*: Chongqing's property tax pilot promoted factor input and economic growth but reduced TFP, strengthening the previous extensive growth pattern relying on factor input*.
H3*: Shanghai's property tax pilot promoted TFP but reduced factor input and economic growth, contributing to the intensive growth pattern relying on TFP*.

Baiardi et al. (2019), Sethi et al. (2020), Jessica et al. (2020), and Wang et al. (2021) explored the impacts of taxation on economic growth and TFP. Because different taxes have different or even opposite economic effects (Arnold et al., 2011), most studies found it challenging to explain the impacts of property taxes on growth patterns. Arnold et al. (2011), Stähler (2019), and Bielecki and Stähler (2022) found that property taxes positively impact the economic growth and social welfare. Surging house prices together with property taxes are a potentially desirable option to finance the sustainable growth of developing countries (Awasthi et al., 2021). Banzhaf et al. (2021) found that property taxes decrease housing consumption, which might hinder economic growth. Bai et al. (2014) and Du and Zhang (2015) found that the economic outcomes of different property tax policies were diametrically opposite. This study explores the effects and underlying mechanisms of policy experiments on real estate in Shanghai and Chongqing in an attempt to reconcile the conflicting evidence collected in the literature.

## Model, Method, and Data

This empirical study investigates the impacts of property taxes in Shanghai and Chongqing on growth patterns by evaluating their effects on economic growth and TFP. According to the neoclassical growth theory, economic growth depends on factor input and TFP. If property taxes improved economic growth but reduced TFP, this growth pattern is extensive and depends on factor input. Intensive growth can be encouraged by relying on TFP.

### Model and Method

As China has not imposed property taxes on a national scale, we cannot directly use data on property taxes for multiple regression analysis. Local governments may also choose different tax rates according to local economic situations, which will cause reverse causality. In addition, multiple regression analysis may omit important variables. These problems will bias estimations.

The property tax pilots in Chongqing and Shanghai provide an opportunity to evaluate the impacts of property taxes on growth patterns. The central government determined the specific cities and times of the pilots, which were exogenous for each city. The pilots in Chongqing and Shanghai act as a quasi-natural experiment to which we applied difference-in-differences to measure the effects on economic growth and TFP.

The pilot cities are the treatment group while other cities are included as a control. Under the hypothesis that the two groups are subjected to common trends, any differences between the trends before and after treatment can likely be attributed to property taxes. Difference-in-differences estimation effectively avoids the reverse causality and the omission of essential variables. By estimating the different impacts of the property tax pilots between the pilot cities and other cities, we can identify the causal relationships between property taxes, economic growth, and TFP. The relevant regression formula is as follows:


(2) $$\begin{array}{l} outcome_{it}=\beta_{0}+\beta_{1}{pilot_{i}^{*}after}_{t}+\beta_{2}X_{it}+\varepsilon_{i}+\omega_{t}+\xi_{it}. \end{array}$$

where the variable *outcome*_*it*_ is the explained variable. *Pilot*_*i*_ is an indicator variable; if the city belongs to the pilot cities, it is set to 1; otherwise, it is set to 0. *After*_*t*_ is also an indicator variable; before the launch of the pilots, the variable is set to 0; otherwise, it is set to 1. *X*_*it*_ is a group of control variables affecting the economic growth and TFP; ε_*i*_is the city fixed effect; ω_*t*_ is the year fixed effect; and ξ_*it*_ is the error term. *Pilot*_*i*_ and *after*_*t*_ are included in ε_*i*_ and ω_*t*_, respectively.

### Variable Definitions

#### Explained Variables

The explained variables include economic growth measured by the natural logarithm of real GDP per capita and TFP. Based on the study by Griliches and Mairesse (1991), we used the following equation to estimate TFP:


(3) $$\begin{array}{l} TFP_{it}=lnY_{it}/L_{it}-\alpha lnK_{it}/L_{it} \end{array}$$

*where Y*_*it*_ represents the aggregate output of city *i* in year *t*. *L*_*it*_ represents the labor input of city *i* in year *t*, measured by the number of employees. *K*_*it*_ represents the capital stock of city *i* in year *t*. The parameter α represents the output elasticity for capital; from Hall (1999), α =1/3. Following the trend of previous studies, we used the perpetual inventory method to calculate the capital stock of each city, as follows:


(4) $$\begin{array}{l} K_{it}=I_{it}+\left(1-\delta \right)K_{it-1}. \end{array}$$

*I*_*it*_ is the capital input of city *i* in year *t*, represented by real fixed asset investment. δ is the depreciation rate; from Zhang (2008), δ = 9.6%. The base year is 2000, and the initial capital stock calculation method is drawn from Reinsdorf and Cover (2005). The third explained variable of this study is house prices.

#### The Core Explanatory Variable

The interactive term in Equation (2), namely ${pilot_{i}^{*}after}_{t}$, is the core explanatory variable of this study. Its coefficient, β_1_, represents the net effects of the property tax pilots on the explained variables.

#### Control Variables

The control variables include the following: investment intensity measured by the natural logarithm of total fixed asset investment per capita; the development level of the secondary industry measured by the proportion of the secondary sector in GDP; the development level of the service industry measured by the ratio of the tertiary industry in GDP; fiscal policy measured by the natural logarithm of fiscal spending per capita; labor input measured by the proportion of employees in the total population; and openness measured by the natural logarithm of actual foreign direct investment per capita. When examining the impacts of the property tax pilots on economic growth, TFP acts as a control variable. The real GDP per capita also acts as a control variable when investigating the effects of the property tax pilots on TFP and house prices.

### Data Sources

The panel data of 256 cities from 2005 to 2016 are from the *China Statistical Yearbook for Regional Economy, China City Statistical Yearbook*, and *China Statistical Yearbook*. Variable definitions and descriptive statistics are shown in Table 2.

**Table 2:** Variable definitions and descriptive statistics.

**Variable**	**Definitions**	**Obs**	**Mean**	**Std. Dev**.
Real GDP per capita	Natural logarithm of real GDP per capita	2,945	10.216	0.806
TFP	Total factor productivity	2,945	9.900	0.318
Investment	Natural logarithm of total fixed assets investment per capita	2,945	9.723	0.927
Secondary industry	Proportion of secondary sector in GDP	2,945	0.492	0.104
Tertiary industry	Proportion of tertiary sector in GDP	2,945	0.372	0.087
Fiscal policy	Natural logarithm of fiscal spending per capita	2,945	8.267	0.818
Labor	Proportion of employees in total population	2,945	0.120	0.115
Openness	Natural logarithm of foreign direct investment per capita	2,945	3.910	1.711

## Results

### Baseline Regression Results

Equation (2) is used to investigate the impacts of the property tax pilots on economic growth and TFP to verify Hypotheses 2–3. Table 3 presents the results. The regression model (1) estimates the effect of the pilots on economic growth, and no significant impact was found. Hypotheses 2 and 3 postulate that the property taxes in Shanghai and Chongqing had different effects on economic growth. Therefore, regression models (2) and (3) investigate the impacts of the pilots of Chongqing and Shanghai on economic growth, respectively. We found that Chongqing's pilot had a significant positive impact on economic growth, while the pilot in Shanghai had a significant negative effect. Specifically, Chongqing's property tax pilot increased GDP per capita by 24.6% (e^0.22^-1 = 0.246), and Shanghai's property tax pilot reduced GDP per capita by 7.5% (e^0.072^-1 = 0.075).

**Table 3:** Baseline regression results.

**Variables**	**Economic growth**			**TFP**		
	**(1)**	**(2)**	**(3)**	**(4)**	**(5)**	**(6)**
	**Shanghai, Chongqing**	**Chongqing**	**Shanghai**	**Shanghai, Chongqing**	**Chongqing**	**Shanghai**
${\text{Pilot}}_{\text{i}}^{*}$after_t_	0.075 (0.106)	0.220*** (0.035)	−0.072* (0.039)	−0.080 (0.136)	−0.266*** (0.033)	0.107** (0.043)
TFP	0.715*** (0.123)	0.719*** (0.123)	0.721*** (0.122)			
Real GDP per capita				0.893*** (0.059)	0.893*** (0.059)	0.893*** (0.059)
Investment	0.243*** (0.022)	0.242*** (0.021)	0.242*** (0.021)	−0.273*** (0.015)	−0.271*** (0.015)	−0.271*** (0.015)
Secondary industry	1.031*** (0.120)	1.030*** (0.120)	1.028*** (0.120)	−0.801*** (0.170)	−0.801*** (0.171)	−0.799*** (0.172)
Tertiary industry	0.809*** (0.134)	0.814*** (0.134)	0.808*** (0.135)	−0.813*** (0.177)	−0.820*** (0.177)	−0.814*** (0.178)
Fiscal policy	0.107*** (0.031)	0.104*** (0.032)	0.104*** (0.031)	−0.060*** (0.022)	−0.057** (0.022)	−0.057** (0.022)
Labor	1.375*** (0.312)	1.383*** (0.314)	1.377*** (0.309)	−1.860*** (0.292)	−1.861*** (0.294)	−1.848*** (0.289)
Openness	−0.000 (0.003)	−0.000 (0.003)	−0.000 (0.003)	0.003 (0.003)	0.003 (0.003)	0.003 (0.003)
Constants	−1.202 (1.266)	−1.214 (1.253)	−1.223 (1.250)	4.815*** (0.459)	4.772*** (0.457)	4.764*** (0.459)
City fixed effect	Control	Control	Control	Control	Control	Control
Year fixed effect	Control	Control	Control	Control	Control	Control
Adjusted *R*^2^	0.980	0.981	0.981	0.871	0.873	0.874
*N*	2,945	2,933	2,933	2,945	2,933	2,933

Parentheses indicate robust standard error clustered in city level; ***, **, and * represent the significance levels of 1, 5, and 10%, respectively.

The regression model (4) estimates the impact of the pilots on TFP and no significant impact was found. It is also possible that the pilots in Shanghai and Chongqing had different effects on TFP. Regression models (5) and (6) investigate the impacts of the pilots on the TFP of Chongqing and Shanghai, respectively. We found that Chongqing's pilot had a significant negative effect on TFP, while Shanghai's pilot had a significant positive impact on the TFP. Specifically, the pilot in Chongqing reduced the TFP by 0.266, and the pilot in Shanghai increased the TFP by 0.107.

Neoclassical growth theory purports that economic growth is determined by factor input and TFP. We can therefore surmise that the pilot in Chongqing promoted economic growth by increasing factor input, and the positive effect of increased factor input on economic growth exceeded the negative effect of TFP decline. Therefore, Chongqing's pilot strengthened the previous extensive growth pattern. However, in Shanghai, the positive effect of TFP improvement on economic growth was insufficient to offset the negative impact of reductions in factor input on economic growth. While Shanghai's property tax pilot inhibited economic growth, it transformed the previous extensive growth pattern into an intensive growth pattern.

### Robustness Tests

We performed various tests to confirm the reliability of the above conclusions (refer to the below subsections).

#### Changing Explained Variables

We used the natural logarithm of GDP to measure economic growth and labor productivity measured by the natural logarithm of real GDP/working population as a proxy for TFP. The results presented in Table 4 support the results of baseline regression.

**Table 4:** Replacing explained variables.

**Variables**	**Economic growth**		**TFP**	
	**(1)**	**(2)**	**(3)**	**(4)**
	**Chongqing**	**Shanghai**	**Chongqing**	**Shanghai**
${\text{Pilot}}_{\text{i}}^{*}$after_t_	0.231*** (0.041)	−0.101*** (0.035)	−0.414*** (0.064)	0.115* (0.063)
TFP	0.706*** (0.129)	0.707*** (0.129)		
Real GDP per capita			0.054 (0.038)	0.054 (0.038)
Investment	0.229*** (0.023)	0.229*** (0.024)	−0.008 (0.017)	−0.008 (0.017)
Secondary industry	1.207*** (0.147)	1.205*** (0.147)	−1.392*** (0.216)	−1.389*** (0.217)
Tertiary industry	0.902*** (0.201)	0.895*** (0.201)	−1.471*** (0.288)	−1.462*** (0.288)
Fiscal policy	0.024 (0.023)	0.025 (0.023)	−0.014 (0.024)	−0.015 (0.024)
Labor	1.325*** (0.356)	1.319*** (0.351)	−2.894*** (0.505)	−2.876*** (0.498)
Openness	−0.002 (0.003)	−0.002 (0.003)	0.008 (0.005)	0.008 (0.005)
Constants	−3.820*** (1.363)	−3.833*** (1.359)	−0.039 (0.412)	−0.046 (0.413)
City fixed effect	Control	Control	Control	Control
Year fixed effect	Control	Control	Control	Control
Adjusted *R*^2^	0.980	0.980	0.761	0.756
*N*	2,933	2,933	2,935	2,935

Parentheses indicate robust standard error clustered in city level; *** and * represent the significance levels of 1% and 10%, respectively.

#### Changing Time Window

The difference-in-differences estimation using data over multiple periods may cause series-autocorrelation and bring in confounding factors, resulting in biased estimations (Bertrand et al., 2004). Therefore, this study retains 2 years of data around 2011 and uses difference-in-differences in re-estimation. The results reported in Table 5 show that series-autocorrelation and time window selection did not exert significant effects.

**Table 5:** Changing time window.

**Variables**	**Economic growth**		**TFP**	
	**(1)**	**(2)**	**(3)**	**(4)**
	**Chongqing**	**Shanghai**	**Chongqing**	**Shanghai**
${\text{Pilot}}_{\text{i}}^{*}$after_t_	0.045*** (0.006)	−0.103*** (0.023)	−0.018** (0.008)	0.206*** (0.042)
TFP	0.354*** (0.098)	0.354*** (0.098)		
Real GDP per capita			0.777*** (0.073)	0.777*** (0.073)
Investment	0.122*** (0.030)	0.122*** (0.030)	−0.160*** (0.026)	−0.160*** (0.026)
Secondary industry	1.715*** (0.452)	1.715*** (0.452)	−0.995** (0.412)	−0.995** (0.412)
Tertiary industry	0.502 (0.598)	0.502 (0.598)	−0.816** (0.393)	−0.816** (0.393)
Fiscal policy	0.012 (0.024)	0.012 (0.024)	−0.030 (0.025)	−0.030 (0.025)
Labor	1.773*** (0.531)	1.773*** (0.531)	−4.633*** (0.612)	−4.633*** (0.612)
Openness	0.008* (0.005)	0.008* (0.005)	−0.012** (0.006)	−0.012** (0.006)
Constants	4.118*** (1.574)	4.122*** (1.575)	5.158*** (0.719)	5.160*** (0.719)
City fixed effect	Control	Control	Control	Control
Year fixed effect	Control	Control	Control	Control
Adjusted *R*^2^	494	494	494	494
*N*	0.962	0.962	0.861	0.861

Parentheses indicate robust standard error clustered in the city level; ***, **, and * represent the significance levels of 1, 5, and 10%, respectively.

#### Controlling Province × Year Fixed Effect

We controlled the province × year fixed effect to exclude the influences of provincial time-varying factors. The results presented in Table 6 support the conclusions drawn in the previous sub-section.

**Table 6:** Controlling province × year effect.

**Variables**	**Economic growth**		**TFP**	
	**(1)**	**(2)**	**(3)**	**(4)**
	**Chongqing**	**Shanghai**	**Chongqing**	**Shanghai**
${\text{Pilot}}_{\text{i}}^{*}$after_t_	0.349*** (0.044)	−0.156** (0.064)	−0.298*** (0.075)	0.218*** (0.083)
TFP	0.708*** (0.137)	0.708*** (0.137)		
Real GDP per capita			0.914*** (0.058)	0.914*** (0.058)
Investment	0.256*** (0.034)	0.256*** (0.034)	−0.313*** (0.019)	−0.313*** (0.019)
Secondary industry	0.860*** (0.187)	0.860*** (0.187)	−0.592** (0.231)	−0.592** (0.231)
Tertiary industry	0.439** (0.173)	0.439** (0.173)	−0.399* (0.218)	−0.399* (0.218)
Fiscal policy	0.104*** (0.037)	0.104*** (0.037)	−0.079*** (0.029)	−0.079*** (0.029)
Labor	1.313*** (0.301)	1.313*** (0.301)	−1.819*** (0.271)	−1.819*** (0.271)
Openness	0.002 (0.003)	0.002 (0.003)	−0.000 (0.003)	−0.000 (0.003)
Constants	−0.989 (1.541)	−0.976 (1.540)	4.838*** (0.504)	4.835*** (0.504)
City fixed effect	Control	Control	Control	Control
Year fixed effect	Control	Control	Control	Control
Province × year fixed effect	Control	Control	Control	Control
Adjusted *R*^2^	2,933	2,933	2,933	2,933
*N*	0.994	0.984	0.892	0.891

Parentheses indicate robust standard error clustered in city level; ***, **, and * represent the significance levels of 1, 5, and 10%, respectively.

#### Testing Common Trends

One hypothesis of difference-in-differences is that the treatment and control groups should have common trends before the occurrence of exogenous shocks. However, in baseline regressions, this premise has not been confirmed. Drawing from Moser and Voena (2012), we used the following equation to test for common trends:


(5) $$\begin{array}{l} outcome_{it}=\beta_{0}+\beta_{t}year_{t}^{*}pilot_{i}^{*}pre\;2011_{t}+\beta_{2}X_{it}\\ \;+\varepsilon_{i}+\omega_{t}+\xi_{it} \end{array}$$

In this equation, we allowed β_*t*_ to vary across treatment and control cities before the pilots, with 2008 as the baseline. Table 7 reports the results. The 95% confidence intervals of the treatment and control groups intersect, which indicates that there were no systematic differences in pre-trends across the treatment and control groups. This confirms the validity of difference-in-differences as a methodology for the estimation of the influences of property taxes on growth patterns.

**Table 7:** Pre-pilot time trends in economic growth and TFP by treatment vs. control cities.

**Year dummies**	**Economic growth**			**TFP**		
	**Coefficients**	**[95% Conf. Interval]**		**Coefficients**	**[95% Conf. Interval]**	
Chongqing_2005	−0.038	−0.097	0.021	−0.046	−0.087	−0.004
Chongqing_2006	−0.050	−0.097	−0.003	−0.019	−0.052	0.013
Chongqing_2007	−0.036	−0.063	−0.009	−0.006	−0.024	0.013
Chongqing_2008						
Chongqing_2009	−0.005	−0.017	0.007	−0.001	−0.012	0.011
Chongqing_2010	0.013	−0.016	0.041	0.017	−0.009	0.043
Shanghai_2005	−0.057	−0.147	0.034	−0.071	−0.143	0.001
Shanghai_2006	−0.047	−0.097	0.003	−0.031	−0.057	−0.005
Shanghai_2007	−0.020	−0.041	0.001	−0.013	−0.025	0.000
Shanghai_2008						
Shanghai_2009	0.016	−0.002	0.035	0.004	−0.013	0.020
Shanghai_2010	0.056	0.022	0.090	−0.002	−0.026	0.022
untreated_2005	−0.037	−0.107	0.033	−0.065	−0.112	−0.017
untreated_2006	−0.018	−0.073	0.036	−0.059	−0.096	−0.023
untreated_2007	−0.009	−0.038	0.020	−0.033	−0.052	−0.014
untreated_2008						
untreated_2009	0.011	−0.004	0.026	−0.005	−0.020	0.010
untreated_2010	0.044	0.013	0.075	−0.006	−0.035	0.023

*Standard error was clustered in the city level; 2008 is the baseline*.

We also used propensity score matching difference-in-differences (PSM-DID) to estimate the influences of the property tax pilots. PSM-DID is commonly applied to the problem of non-common trends. This method looks for control cities similar to treatment cities to form a new sample. The kernel function is epacnechnikov, and we specified the logit estimation of the propensity score. Table 8 reports the regression results, which show that the conclusions of baseline regression remain unchanged.

**Table 8:** Using PSM-DID.

**Variables**	**Economic growth**		**TFP**	
	**(1)**	**(2)**	**(3)**	**(4)**
	**Chongqing**	**Shanghai**	**Chongqing**	**Shanghai**
${\text{Pilot}}_{\text{i}}^{*}$after_t_	0.122*** (0.035)	−0.100*** (0.022)	−0.406*** (0.021)	0.157*** (0.036)

Parentheses indicate robust standard error clustered in the city level; ***represents the significance level of 1%. X_it_ and fixed effects were controlled.

#### Placebo Tests

As suggested by Ferrara et al. (2012) and Liu and Lu (2015), we conducted a placebo test to exclude the impacts of other confounding factors on the estimation results. If the pilots were exogenous and not affected by confounding factors, we can directly obtain a consistent estimator of β_1_ through ordinary least-square (OLS) estimation. However, in practice, it is impossible to control for all factors that might affect the explained variables. Therefore, the estimation results are as follows:


(6) $$\begin{array}{l} {\hat{\beta }}_{1}=\beta_{1}+\gamma \frac{cov\left(piloti_{i}\times aftert_{t},\xi_{it}|X_{it}\right)}{var\left(piloti_{i}\times aftert_{t}|X_{it}\right)} \end{array}$$

When γ = 0, unobservable factors do not affect the results, that is, ${\hat{\beta }}_{1}$ is unbiased. As γ is unobservable, this is difficult to test. One approach is to find a random variable that theoretically has no impact on the explained variable to replace *pilot*_*i*_, and then adjust the regression formula as follows:


(7) $$outcome_{it}=\beta_{0}+\beta_{1}randompilot_{i}^{*}after_{t}+\beta_{2}X_{it}+\varepsilon_{i}+\omega_{t}+\xi_{it}\text{​​​​​ }$$

**Figure 1:**
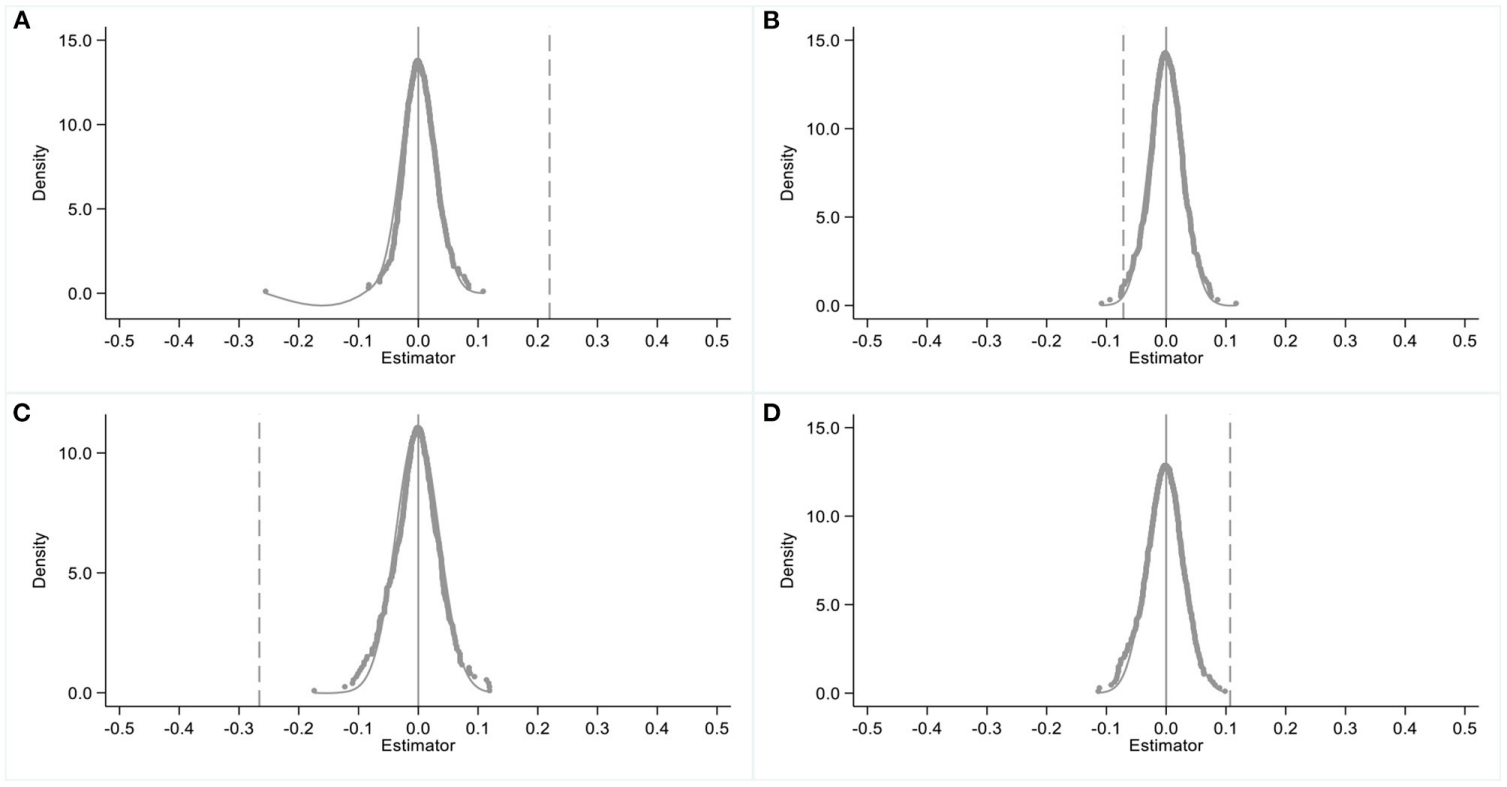
Placebo test I. The impacts of random shocks on the economic growth of Chongqing and Shanghai are estimated in **(A,B)**, respectively, while the impacts of random shocks on the TFP of Chongqing and Shanghai are estimated in **(C,D)**, respectively.

As *randompilot*_*i*_ is a random variable, β_1_ = 0. Thus, if ${\hat{\beta }}_{1}=0$, then γ = 0; otherwise, γ≠0, and the estimation results are biased. This study makes the property tax pilots random and repeats the randomization 500 times to ensure that *randompilot*_*i*_ will not affect *outcome*_*it*_. Figure 1 shows the estimated random distribution of ${\hat{\beta }}_{1}$. Compared with the baseline results, the estimated random distribution is centered around 0 and not significant, as only a few estimators cross the vertical lines. Therefore, we can assume that γ = 0, that is, unobservable factors have a negligible effect.

We carried out another placebo test by advancing the time of the pilots by 1 year. Table 9 reports the regression results. We found that the estimated coefficients of core explanatory variables are not significant. This indicates that it was indeed the pilots which produced the effects recorded in the baseline regression results rather than other factors.

**Table 9:** Placebo test II.

**Variables**	**Economic growth**		**TFP**	
	**(1)**	**(2)**	**(3)**	**(4)**
	**Chongqing**	**Shanghai**	**Chongqing**	**Shanghai**
${\text{Pilot}}_{\text{i}}^{*}$after_t_	−0.026 (0.031)	−0.021 (0.029)	0.049 (0.038)	0.043 (0.036)
TFP	0.716*** (0.124)	0.720*** (0.122)		
Real GDP per capita			0.891*** (0.060)	0.894*** (0.059)
Investment	0.241*** (0.021)	0.243*** (0.021)	−0.270*** (0.015)	−0.272*** (0.014)
Secondary industry	1.034*** (0.120)	1.027*** (0.120)	−0.802*** (0.171)	−0.798*** (0.171)
Tertiary industry	0.819*** (0.134)	0.805*** (0.135)	−0.827*** (0.177)	−0.810*** (0.178)
Fiscal policy	0.106*** (0.032)	0.104*** (0.031)	−0.059*** (0.023)	−0.058*** (0.022)
Labor	1.391*** (0.322)	1.373*** (0.307)	−1.878*** (0.303)	−1.845*** (0.287)
Openness	0.000 (0.003)	−0.000 (0.003)	0.002 (0.003)	0.003 (0.003)
Constants	−1.198 (1.264)	−1.226 (1.253)	4.803*** (0.463)	4.777*** (0.456)
City fixed effect	Control	Control	Control	Control
Year fixed effect	Control	Control	Control	Control
Adjusted *R*^2^	0.980	0.981	0.871	0.873
*N*	2,933	2,933	2,933	2,933

Parentheses indicate robust standard error clustered in the city level; ***represents the significance level of 1%.

#### The Panel Data Approach for Program Evaluation Suggested by Hsiao et al. (2012)

**Figure 2:**
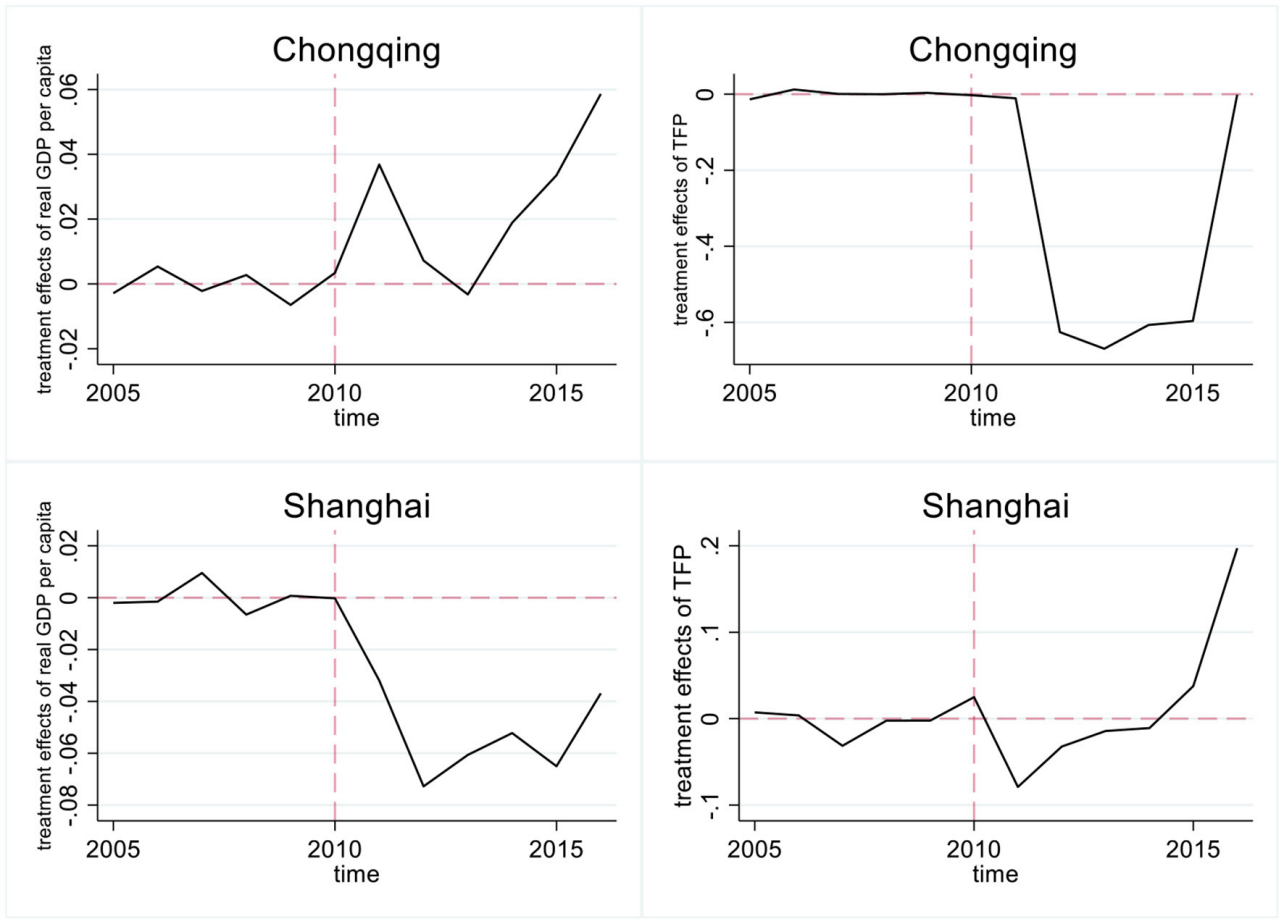
Results of panel data approach for program evaluation suggested by Hsiao et al. (2012).

In addition to baseline regression, we used an alternative method to estimate the effects of property taxes on growth patterns. Hsiao et al. (2012) proposed the panel data approach for program evaluation, which is well-suited to cases involving only a few individuals or regions and can effectively avoid selection bias. This method of causal inference has become popular in recent years. We used the approach to estimate hypothetical economic growth and TFP without property taxation for Shanghai and Chongqing. By calculating the difference between actual as well as hypothetical economic growth and TFP, we can determine the treatment effect of the pilots on economic growth and TFP in Chongqing and Shanghai. The results are presented in Figure 2.

The hypothetical and actual data are similar before the pilots, which confirms the feasibility of this method. Moreover, most of our research conclusions remain unchanged. However, the pilot in Shanghai decreased TFP in the first few years after the pilot, which might be because, the pilot slowed economic growth (which has a positive impact on TFP). With the transformation of the growth pattern, TFP rebounded rapidly. Therefore, the estimation results still indicate that, on average, Shanghai's property tax pilot positively impacted its TFP.

#### Standard Error Correction

In the quasi-natural experiment of this study, there are only two cities in the treatment group; ignoring this aspect of the data can lead to standard errors. Following the approach suggested by Anukriti (2018), we therefore aggregated the data to the province-year level. The standard errors from this grouped estimation are likely to be more reliable (Angrist and Pischke, 2009). Table 10 reports the regression results, which support the conclusions of baseline regression.

**Table 10:** Standard error correction.

**Variables**	**Economic growth**		**TFP**	
	**(1)**	**(2)**	**(3)**	**(4)**
	**Chongqing**	**Shanghai**	**Chongqing**	**Shanghai**
${\text{Pilot}}_{\text{i}}^{*}$after_t_	0.191*** (0.056)	−0.126* (0.073)	−0.221*** (0.055)	0.173** (0.065)
TFP	0.768*** (0.069)	0.773*** (0.066)		
Real GDP per capita			0.803*** (0.079)	0.802*** (0.080)
Investment	0.235*** (0.022)	0.236*** (0.022)	−0.239*** (0.035)	−0.240*** (0.035)
Secondary industry	1.468*** (0.166)	1.472*** (0.174)	−1.080*** (0.264)	−1.085*** (0.274)
Tertiary industry	1.429*** (0.259)	1.430*** (0.266)	−1.275*** (0.278)	−1.275*** (0.292)
Fiscal policy	0.087* (0.050)	0.086* (0.050)	0.003 (0.034)	0.004 (0.034)
Labor	1.760*** (0.497)	1.721*** (0.480)	−2.152*** (0.426)	−2.087*** (0.413)
Openness	0.003 (0.005)	0.002 (0.005)	−0.002 (0.007)	−0.001 (0.007)
Constants	−1.979** (0.758)	−2.016** (0.732)	5.259*** (0.614)	5.262*** (0.622)
City fixed effect	Control	Control	Control	Control
Year fixed effect	Control	Control	Control	Control
Adjusted *R*^2^	0.995	0.995	0.952	0.952
*N*	299	299	299	299

Parentheses indicate robust standard error clustered in the city level; *** and * represent significance levels of 1% and 10%, respectively.

## Discussion

There are several notes worth making with regard to these results. First, property buyers in Chongqing and Shanghai would likely have known about the property tax pilots before they were implemented. However, any behavior resulting from this anticipation would only lead to a downward bias in the regression results and would not therefore affect the research conclusions. Second, buyers in Chongqing and Shanghai could move to other cities to buy houses, biasing the estimation results. However, it is not easy to buy homes outside of one's permanent residence in China. For example, there are restrictions on household registration, work, and insurance. Third, economic growth may have a spatial correlation. However, even if this was the case, it would only cause downward bias and not affect the research conclusions. The above robustness tests show that the research conclusions of this study are highly reliable.

### Underlying Mechanisms

Our theoretical analysis indicated that the mechanisms through which property taxes affect growth patterns involve house prices. Therefore, it is necessary to investigate the impacts of property taxes on house prices in Shanghai and Chongqing to verify Hypothesis 1. As data on house prices were only updated up to 2013, we only retained the data 3 years before and after 2011. The estimation results are presented in Table 11, the pilot in Chongqing increased house prices, while the pilot in Shanghai reduced house prices.

**Table 11:** Mechanism.

**Variables**	**Average house price**		**Ordinary house prices**	
	**(1)**	**(2)**	**(3)**	**(4)**
	**Chongqing**	**Shanghai**	**Chongqing**	**Shanghai**
${\text{Pilot}}_{\text{i}}^{*}$after_t_	0.026* (0.016)	−0.104*** (0.015)	0.111*** (0.026)	−0.129*** (0.028)
Real GDP per capita	−0.004 (0.078)	−0.003 (0.078)	−0.033 (0.060)	−0.015 (0.067)
Investment	−0.017 (0.024)	−0.019 (0.024)	−0.000 (0.063)	−0.014 (0.063)
Secondary industry	1.771*** (0.333)	1.762*** (0.334)	−0.005 (0.020)	−0.012 (0.021)
Tertiary industry	1.633*** (0.394)	1.637*** (0.395)	−0.008 (0.021)	−0.012 (0.022)
Fiscal policy	0.009 (0.026)	0.008 (0.026)	−0.017 (0.040)	−0.012 (0.041)
Labor	−0.183* (0.099)	−0.186* (0.101)	0.108 (0.163)	0.057 (0.178)
Openness	−0.010 (0.008)	−0.009 (0.008)	−0.009 (0.021)	−0.009 (0.021)
Constants	−2.724*** (0.737)	−2.704*** (0.739)	0.279 (1.857)	0.746 (1.892)
City fixed effect	Control	Control	Control	Control
Year fixed effect	Control	Control	Control	Control
Adjusted *R*^2^	0.862	0.861	0.884	0.879
*N*	1,481	1,481	202	202

Parentheses indicate robust standard error clustered in the city level; *** and * represent significance levels of 1 and 10%, respectively.

Bai et al. (2014) also drew similar conclusions. Their hypothesis was consistent with Hypothesis 1, which postulates that the increase in Chongqing's house prices was because, property taxes increased the prices of ordinary houses, while Shanghai's pilot reduced the prices of ordinary houses. However, Bai et al. (2014) did not conduct empirical research on the relationship between property taxes and the prices of different housing. Verifying Hypothesis 1 requires investigating the impacts of the pilots on ordinary house prices; however, it is not possible to obtain statistics on ordinary house prices in each city. Only 35 large- and medium-sized cities have statistics on average house prices and high-end prices. We therefore estimated ordinary house prices using the following formula: (sales of houses – sales of high-end houses) / (sales area of houses – sales area of high-end houses). We used the natural logarithm of the ordinary house prices as explained variables, and the estimation results are reported in Regression Models (3) and (4) of Table 11.

These empirical results indicate heterogeneous effects, the pilot of Chongqing increased ordinary house prices, while that of Shanghai reduced ordinary house prices. Therefore, the property tax pilots created opposite effects. Due to the narrow tax base, the pilot of Chongqing triggered the substitution effect between high-end houses and ordinary houses; thus, property taxes in Chongqing increased house prices and undermined intensive growth. The pilot of Shanghai reduced house prices and prompted intensive growth.

## Conclusions and Policy Implications

Based on the panel data of 256 cities from 2005 to 2016, this study uses difference-in-differences, propensity score matching difference-in-differences, and the panel data approach for program evaluation suggested by Hsiao et al. (2012) to investigate the impacts of the property tax pilots in Chongqing and Shanghai on growth patterns. As Chongqing's property taxes have been mainly for high-end houses rather than ordinary houses, those who originally wanted high-end houses might have purchased ordinary houses, thus increasing the average house price. The pilot of Chongqing therefore promoted economic growth but significantly decreased the TFP, strengthening the previous extensive growth pattern which relies on factor input. Shanghai's property taxes reduced house prices and slowed economic growth but increased the TFP, which promoted intensive growth (which relies on TFP).

Since the reform and opening up of China, the economy has mainly relied on factor input to promote economic growth. However, this pattern of extensive growth causes problems such as low investment efficiency, environmental pollution, financial risks, and infringements on workers' rights. Because of the disappearance of demographic dividends and the law of diminishing marginal return on capital, China urgently needs to transform its growth pattern. The empirical evidence of this study indicates that the Shanghai's property tax pilot is the one to learn from, while Chongqing's policies should not be repeated.

The findings of this study have the following specific policy implications. First, China should adhere to property tax legislation. This study shows that as long as policies are appropriate, property taxes can restrain house prices and achieve intensive growth by improving TFP. High-quality development has become a goal of the Chinese government's macroeconomic policies. Within this context, we recommend adhering to property tax legislation to promote high-quality development through intensive growth. Second, the purpose of the pilots was not to increase house prices; therefore, China should carefully formulate policy with the narrow tax base in mind, to avoid incurring the substitution effect. Third, China should perfect property tax policies to increase TFP when the economy is stable or booming, which helps to achieve intensive growth without causing an economic downturn. More recently, the economy of Mainland China has been severely affected by the COVID-19 pandemic. China's Ministry of Finance has stated that due to current economic conditions, property tax should not be levied in 2022. This is consistent with our suggestions. However, some cities have released administrative restrictions on the real estate markets. This may further push up house prices and strengthen the past extensive growth pattern. Finally, other developing countries can learn from China's experience and take advantage of the real estate market to promote economic growth in the early stages of national economic development. They can also draw on China's experience in property tax reform to regulate the real estate market and promote intensive growth.

## Data Availability Statement

The data analyzed in this study is subject to the following licenses/restrictions: used data is from the China Statistical Yearbook for Regional Economy, China City Statistical Yearbook, and China Statistical Yearbook. Requests to access these datasets should be directed to https://data.cnki.net/area/yearbook/Single/N2005110340?dcode=D08.

## Author Contributions

HZ: guarantor of the integrity of the entire study, study concept and design, literature research, manuscript preparation, and manuscript editing. SL: data analysis and statistical analysis. Both authors contributed to the article and approved the submitted version.

## Funding

This research was funded by the Science Foundation of Zhejiang Sci-Tech University (ZSTU) under Grant No. 20092343-Y.

## Conflict of Interest

The authors declare that the research was conducted in the absence of any commercial or financial relationships that could be construed as a potential conflict of interest.

## Publisher's Note

All claims expressed in this article are solely those of the authors and do not necessarily represent those of their affiliated organizations, or those of the publisher, the editors and the reviewers. Any product that may be evaluated in this article, or claim that may be made by its manufacturer, is not guaranteed or endorsed by the publisher.
